# Genome wide identification of serotonin N-acetyltransferase (*PbSNAT*) gene family and their role in pear (*Pyrus bretschneideri)* fruit development

**DOI:** 10.3389/fpls.2026.1767313

**Published:** 2026-03-02

**Authors:** Chen Chen, Xiangyu Zuo, Aneesa Gul, Shunyan Chen, Ahmad Ali

**Affiliations:** 1 Shanxi Datong University, Medical School of Shanxi Datong University, Datong, China; 2 School of Traditional Chinese Medicine, Shanxi Datong University, Datong, Shanxi, China; 3 College of Coastal Agricultural Sciences, Guangdong Ocean University, Zhanjiang, China; 4 State Key Laboratory of Tropical Crop Breeding, Key Laboratory of Biology and Genetic Resources of Tropical Crops Ministry of Agriculture and Rural Affairs, Key Laboratory of Conservation and Utilization of Tropical Agricultural Biological Resources of Hainan Province, Institute of Tropical Bioscience and Biotechnology, Chinese Academy of Tropical Agricultural Sciences, Haikou, China

**Keywords:** bioinformatic analysis, fruit development, gene family, *PbSNAT*, pear

## Abstract

**Introduction:**

Serotonin N-acetyltransferase (SNAT) is a key enzyme catalyzing a critical step in phytomelatonin biosynthesis, which plays important roles in plant growth and stress responses. However, the SNAT gene family has not been systematically characterized in Pyrus bretschneideri, an economically important pear species. This study aims to fill this gap by conducting a comprehensive analysis of the *PbSNAT* gene family and exploring its functional implications.

**Methods:**

A genome-wide identification of *PbSNAT* genes was performed in *P. bretschneideri*. Phylogenetic analysis was used to classify *PbSNAT* proteins into distinct clades. Physicochemical properties, motif compositions, and gene structures of *PbSNATs* were analyzed to evaluate their conservation. Promoter and Gene Ontology (GO) analyses were conducted to predict their regulatory mechanisms and functional roles. Expression profiling of *PbSNAT* genes was carried out across different tissues and fruit developmental stages of five pear cultivars to investigate their expression patterns. Additionally, the correlation between SNAT expression, melatonin (MT) abundance, and fruit development was analyzed.

**Results:**

A total of 51 *PbSNAT* genes (*PbSNAT1–51*) were identified from the pear genome. Phylogenetic analysis grouped these *PbSNAT* proteins into six clades. Most *PbSNATs* are hydrophobic and localized in chloroplasts. Motif and gene structure analyses revealed high conservation within each clade, supporting the phylogenetic classification. Promoter and GO analyses indicated that *PbSNAT* genes are responsive to stress and involved in chloroplast development and indole acetic acid metabolism. Expression profiling showed tissue-specific expression patterns of *PbSNAT* genes, suggesting their roles in organ development. Furthermore, *PbSNAT* genes are widely expressed across five pear cultivars at different fruit developmental stages. *PbSNAT1* was strongly expressed during fruit development, implying its role in fruit setting and ripening. High abundances of SNAT and MT were detected in pericarp and pulp tissues, and their levels were correlated with *PbSNAT46* expression.

**Discussion:**

This study provides the first comprehensive characterization of the PbSNAT gene family in *P. bretschneideri*. The conservation of sequence, structure, and expression patterns of *PbSNATs* suggests their conserved and divergent functional roles. The tissue-specific and fruit development-associated expression of *PbSNAT* genes, along with their correlation with MT abundance, indicates that they play crucial roles in pear growth and fruit development. Particularly, *PbSNAT1* and *PbSNAT46* may be key regulators of fruit setting, ripening, and MT biosynthesis in pear. These findings establish a valuable foundation for future functional studies and molecular breeding of pear.

## 1 Introduction

Melatonin (MT) (N-acetyl-5-methoxytryptamine), a neurohormone, was first isolated in 1958 from the pineal gland of cattle (Sharif et al., 2018). This discovery prompted extensive research into (MT) presence and functions across animal species (Sharif et al., 2018). The presence of MT in higher plants was first established in 1995 through the concurrent work of two independent research groups (Dubbels et al., 1995; Hattori et al., 1995). According to (Sun et al., 2015), MT has been found in more than 20 plant families, which indicates that it has a broad biological influence. Functionally analogous to auxins, MT is considered a phytohormone, stimulating plant growth with 10–55% of the activity of IAA (Arnao and Hernández-Ruiz, 2015). The antioxidant capacity of MT is significantly enhanced by its efficacy in scavenging ROS/RNS and various free radicals (Reiter et al., 2015). Numerous abiotic and biotic stressors, including aging, can boost MT biosynthesis, according to multiple studies (Sharif et al., 2018). Notably, the application of exogenous MT improves plant cold tolerance via several distinct pathways. Some examples of these processes are increasing the activity of antioxidant enzymes (Marta et al., 2016) and activating genes that respond to cold (Turk et al., 2014).

Pears are a nutritious fruit with a pleasant sweet-tart flavor. Cultivated for over 3,000 years, and remain an economically significant crop grown across wide geographic regions (Sheng et al., 2023). Pears are commonly consumed fresh, canned, or processed. Notably, they are also used in dietary therapies, with some studies suggesting potential benefits in mitigating the effects of air pollutants like PM2.5 and supporting immune health against illnesses such as COVID-19. To improve pear fruit quality and production, a deeper genetic understanding is essential. The sequencing of the pear genome enables precise gene prediction and annotation (Chen et al., 2024; Liu et al., 2025).

Recent research indicates that chloroplasts and mitochondria serve as the main sites of MT synthesis in plants (Tan et al., 2013). The four enzymes ASMT, SNAT, T5H, and TDC assist the successive enzymatic transformations that convert tryptophan into MT (Arnao and Hernández-Ruiz, 2015; Wang et al., 2016). However, there has been a lack of rigorous research into the genetic basis of MT synthesis in different crops. Several examples, including Arabidopsis (Byeon et al., 2016), apples (Xia et al., 2020), rice (Byeon et al., 2014), and cassava (Xia et al., 2020), have experimentally confirmed the biological functions of these structural genes. Prominently, TDC and ASMT gene families are commonly found in plant genomes. Particular gene families are known to be integral to how plants cope with environmental and pathogenic stresses (Debnath et al., 2019; Ahmed et al., 2020; Sharif et al., 2026b). In a recent study, the tomato genes *SlASMT03* and *SlASMT07* were shown to exhibit resistance against various pathogenic bacteria (Liu et al., 2017). It has also been reported that the pepper gene *CaASMT06* is induced by cold stress. In apple, the expression of *MdASMT11* and *MdASMT14* increases significantly in response to both cold and salt stress (Wang et al., 2022). SNATs are implicated in the abiotic stress response and are postulated to regulate steady-state MT levels (Park et al., 2014). Research indicates that SNAT enzymes from different species have distinct thermal optima, such as 70 °C in cyanobacteria, 55 °C in loblolly pine, and 35 °C in apple (Zhang et al., 2020). Ectopic expression of *MzSNAT5* in Arabidopsis enhanced drought tolerance, a phenotype associated with increased MT (Wang et al., 2017). Suppressing SNATs in rice lowers MT levels, resulting in a suite of phenotypes including stunted growth, yield penalty, heightened stress vulnerability, and abnormal coleoptile elongation under anoxia (Byeon and Back, 2016). Suppression of *GhSNAT1*, a key gene in MT biosynthesis, impaired cotton innate immunity and abiotic stress tolerance, enhancing vulnerability to pathogens and environmental challenges (Li et al., 2019). Together, these findings emphasize the need to clarify the molecular mechanisms of MT biosynthesis and function in pear, which will enhance understanding of stress tolerance and assist in the genetic improvement of this significant fruit crop.

In this study, we identified 51 *PbSNAT* genes from the Chinese white pear (*Pyrus bretschneideri*) genome. Phylogenetic and sequence analyses elucidated the evolutionary relationships among family members, with protein motif and intron–exon structure analyses supporting their classification. RNA-sequencing revealed tissue-specific expression patterns across various pear tissues. Quantification of SNAT and MT was performed at different developmental stages of fruit development. These findings enhance understanding of *PbSNAT* genes in pear reproduction and provide a foundation for identifying candidate genes involved in fruit development.

## 2 Materials and methods

### 2.1 Identification of SNAT genes from the pear genome

Putative SNAT genes were identified in the pear genome via BLASTP search in Ensembl Plants using *A. thaliana* SNAT sequences as queries. Conserved domain validation was then performed on non-redundant candidates using CDD and Pfam (Ahmad et al., 2023b). For each SNAT protein, the molecular weight (MW), isoelectric point (pI), and instability index were calculated using the ProtParam tool. This study used Plant-mPLoc (http://www.csbio.sjtu.edu.cn/bioinf/plant-multi/#) as a tool to determine the subcellular localization of all *PbSNAT* genes and proteins.

### 2.2 Physical location and synteny of SNAT genes

We extracted gff3-files from the *P. bretschneideri* genome database and mapped them to chromosomes using TBtools (Toolbox for biologists) (v0.6655) and determined the *PbSNAT* genes chromosomal distribution (Chen et al., 2023). The following criteria were used to define gene duplication. That included (1) the alignment length required to encompass more than 90% of the longer gene; (2) the aligned region had to have an identity more significant than 90%; and (3) for closely related genes, only one duplication event was considered.

### 2.3 Phylogenetic analysis of SNAT proteins

The *Arabidopsis*, tobacco, and pear SNAT amino acid sequences were utilized to construct a phylogenetic tree. The initial stage was aligning all sequences using Clustal-Omega, a multiple alignment program (Ahmad et al., 2023a; Tan et al., 2023). After that, the results of the Clustal-Omega were used in the IQ-TREE website to estimate the phylogenetic relationships of SNAT by employing the Maximum Likelihood (ML) approach with a total of one thousand bootstrap replicates. Finally, the iTOL (version 5) online tool was used to create the phylogenetic tree of SNAT proteins (Ahmad et al., 2022a).

### 2.4 Gene structure and conserved motif analysis

The *P. bretschneideri* genome database was queried for details regarding the *PbSNAT* gene family, such as accession number, chromosomal location, ORF length, and exon-intron structure. Gene Structure Display Server (http://gsds.cbi.pku.edu.cn/) generated each gene exon, intron, and UTR (untranslated region) distribution patterns. T online MEME tool (http://meme-suite.org/index.html) was used to examine the PbSNAT protein motif. Each sequence must only include one motif instance, with a maximum of one occurrence per site. Ten motifs were discovered, and their breadth may be anywhere from six to one hundred. The TBtools (Toolbox for Biologists) software (v0.6655) was used to visualize these motifs (Chen et al., 2023).

### 2.5 Protein-protein network analysis

To build the network of protein-protein interactions between Pear SNAT, the sequences of all PbSNAT proteins were uploaded to the online STRING v11.5 database. The maximum number of interactors was set to five for the first shell, and for the second shell, it was set to 10. Lastly, Cytoscape v3.8.2 was used to depict the interaction networks.

### 2.6 Gene ontology analysis of SNAT genes

The PbSNAT protein sequences were further analyzed using the Blast2GO tool (Version 2.7.2; http://www.blast2go.com), as described by (Ullah et al., 2022). By following the procedure outlined in previous work by Ahmad et al. (2022a), we successfully classified the sequences into three categories: cellular component, molecular function, and biological process, based on the Gene Ontology (GO) classification.

### 2.7 Promoter analysis of *PbSNAT* genes

Each *PbSNAT* gene upstream region (1500 bp of ATG) in pear was screened using the PlantCARE approach to identify the known *cis*-regulatory elements involved in growth, hormone response, and stress. The last step was to classify the *cis*-regulatory components based on their roles. The Graphpad Prism (v9.5.0) was used for the visualization of the predicted *cis-*elements.

### 2.8 Prediction of 3D protein structures

The three-dimensional structure of the PbSNAT proteins was predicted using the Phyre2 server, following the procedures detailed in (Kelley et al., 2015). In accordance with the procedure described by (Li et al., 2020), water molecules were excluded from the structures using Accelrys Discovery Studio v4.1, and the final structures were visualized with PyMOL (https://pymol.org/).

### 2.9 Prediction of targeted miRNAs

The genomic sequences of all *PbSNAT* genes were compared to miRNA sequences from the psRNA Target Server (https://www.zhaolab.org/psRNATarget/) using the default parameters (Raza et al., 2023). Subsequently, we followed the same procedures as outlined in our previous study by Ahmad et al. (2023a) and performed the interaction analysis using Cytoscape (https://cytoscape.org/). Finally, the visualizations were enhanced using Adobe Illustrator for clarity.

### 2.10 Expression analysis of the *PbSNAT* family members using transcriptomic datasets

The transcriptomic datasets (RNA-seq) published by Zhang et al. (2016) on fruit development and maturation in five key cultivated pear cultivars such as ‘Hosui’ (*P. pyrifolia*), ‘Yali’ (*P. bretschneideri*), ‘Kuerlexiangli’ (*P. sinkiangensis*), ‘Nanguoli’ (*P. ussuriensis*), and ‘Starkrimson’ (*P. communis*), were selected for this study. These cultivars, which represent the primary pear species cultivated globally, and exhibit a range of maturity characteristics. The datasets were analyzed to explore the expression patterns of the PbSNAT family members and its potential correlations with various fruit traits. For the RNA-seq data a total of 35 fruit samples, comprising five species and seven developmental stages, were used. Seven developmental stages were fruit-setting period at 15 days after full blooming (15 DAB, period 1 [S1]), physiological fruit-dropping stage at 30 DAB (period 2 [S2]), fruit rapid enlargement stage at 55 DAB (period 3 [S3]), a month after fruit enlargement stage at 85 DAB (period 4 [S4]), pre-mature stage at 115 DAB (period 5 [S5]), mature stage (duration varies by species, period 6 [S6]), and fruit senescence stage (period 7 [S7]). Gene expression data were processed to generate a heatmap based on log_2_(fold change)-transformed values for each *PbSNAT* family gene, as well as the FPKM (fragments per kilobase of transcript per million fragments mapped) value for each gene. Clustering was performed using the Pearson correlation coefficient and average linkage method. Differential expression was considered significant at |log_2_(fold change)| > 1.5 and *P-value* < *0.005*. All data were processed and visualized using TBtools (Toolbox for Biologists) v0.6655 (Chen et al., 2023).

### 2.11 Extraction and quantification of SNAT and MT by HPLC-MS/MS

The extraction of SNAT and MT was performed following the method of (Aguilera et al., 2014) with minor modifications. Frozen tissue (0.5 g), including pericarp, pulp, fruit seeds, and fruit core were finely ground in liquid nitrogen. SNAT and MT were extracted with 5 mL of chilled chloroform and vortexed vigorously for 2 minutes. The homogenate was centrifuged at 12,000 × g for 15 minutes at 4 °C. The organic phase was collected and evaporated to dryness under a gentle stream of nitrogen gas. The dried residue was reconstituted in 200 µL of HPLC-grade methanol, filtered through a 0.22 µm PTFE syringe filter, and transferred to an HPLC vial for analysis. All experiments were performed with at least three biological replicates.

### 2.12 Statistical analysis

The statistical analysis was conducted using IBM SPSS Statistics 23 software. The data is presented as the average value plus or minus the standard deviation (SD). A one-way ANOVA was performed on the data, followed by Tukey’s multiple comparison tests. A significant difference was indicated by different lowercase letters above the bars, with a *p*-value of less than 0.05.

## 3 Results

### 3.1 Identification and sequence analysis of *PbSNAT* genes in *P. bretschneideri*

To identify SNAT genes in pear (PbSNATs), we queried the pear genome using the *A. thaliana* (AtSNAT) protein sequence as a reference. After removing redundant sequences, 51 non-redundant *PbSNAT* genes were identified for further analysis (**Table 1**). Their encoded proteins ranged from 145 (PbSNAT23) to 1350 (PbSNAT8 and PbSNAT50) amino acids in length. Correspondingly, the predicted molecular weight (MW) varied from 16.17 kDa (PbSNAT23) to 149.47 kDa (PbSNAT8). The isoelectric points (pI) spanned from 5.41 (PbSNAT39) to 9.73 (PbSNAT17). Subcellular localization predictions indicated distribution across several organelles, including the nucleus, chloroplast, and cell membrane.

**Table 1:** Physiochemical properties of *PbSNAT* gene family in *P. bretschneideri*.

Locus ID	Gene	Chr.	Start	End	No. AA	MW (kDa)	PI	SL
Pbr010685.1	*PbSNAT1*	1	5185113	5186462	239	26.63	5.98	Chloroplast
Pbr013581.1	*PbSNAT2*	1	9700825	9702025	278	31.20	9.24	Cell membrane
Pbr013439.1	*PbSNAT3*	1	10661774	10663610	281	31.80	9.06	Chloroplast
Pbr022924.1	*PbSNAT4*	2	7061472	7062092	212	23.78	8.28	Nucleus
Pbr001058.1	*PbSNAT5*	2	12007267	12011964	603	65.87	8.17	Chloroplast
Pbr027763.1	*PbSNAT6*	2	16339251	16341362	251	28.75	8.31	Nucleus
Pbr041542.1	*PbSNAT7*	2	18000656	18004664	311	35.96	9.44	Nucleus
Pbr025831.1	*PbSNAT8*	3	1873361	1880030	1350	149.47	5.94	Nucleus
Pbr012955.1	*PbSNAT9*	3	2885439	2889049	180	21.09	8.92	Chloroplast
Pbr040444.1	*PbSNAT10*	3	14148181	14151526	402	46.06	9.44	Chloroplast
Pbr013032.1	*PbSNAT11*	3	23069307	23071543	396	45.34	8.90	Chloroplast
Pbr013019.1	*PbSNAT12*	3	23205455	23212053	367	41.65	6.06	Nucleus
Pbr020949.2	*PbSNAT13*	4	6017615	6023671	823	91.27	6.14	Nucleus
Pbr028329.1	*PbSNAT14*	6	2638856	2641340	269	30.39	8.74	Chloroplast
Pbr014573.1	*PbSNAT15*	6	23065341	23066667	159	17.79	5.96	Chloroplast
Pbr009679.1	*PbSNAT16*	7	1563294	1569499	206	23.26	8.01	Cytoplasm
Pbr002893.1	*PbSNAT17*	7	12221602	12222844	276	30.16	9.73	Chloroplast
Pbr039966.2	*PbSNAT18*	7	15131040	15137235	595	66.88	8.85	Vacuole
Pbr008543.1	*PbSNAT19*	8	2371443	2377488	451	52.21	6.66	Nucleus
Pbr032624.1	*PbSNAT20*	9	3919670	3920760	169	19.26	9.16	Chloroplast
Pbr038776.1	*PbSNAT21*	9	22410118	22411125	217	24.33	5.69	Chloroplast
Pbr009075.1	*PbSNAT22*	10	10130527	10134778	556	61.67	6.17	Nucleus
Pbr041950.1	*PbSNAT23*	10	16363776	16364369	145	16.17	9.05	Chloroplast
Pbr008959.1	*PbSNAT24*	11	1655795	1658146	180	21.05	8.19	Chloroplast
Pbr038223.1	*PbSNAT25*	11	4326447	4333154	1319	145.59	5.83	Nucleus
Pbr025703.1	*PbSNAT26*	11	9479572	9481959	269	29.97	8.77	Chloroplast
Pbr025702.1	*PbSNAT27*	11	9526620	9529007	269	29.97	8.77	Chloroplast
Pbr011617.1	*PbSNAT28*	11	25779678	25784102	592	66.64	6.91	Chloroplast
Pbr033752.1	*PbSNAT29*	11	26313515	26318076	592	66.61	6.71	Chloroplast
Pbr028480.2	*PbSNAT30*	12	250701	252667	238	25.98	7.24	Chloroplast
Pbr008272.1	*PbSNAT31*	12	9573133	9574727	376	41.98	9.11	Nucleus
Pbr041797.1	*PbSNAT32*	12	14185802	14191569	667	73.40	8.25	Nucleus
Pbr016744.1	*PbSNAT33*	12	18997060	18999458	394	43.78	8.98	Nucleus
Pbr011953.1	*PbSNAT34*	13	9731362	9732901	398	44.86	5.73	Chloroplast
Pbr025815.1	*PbSNAT35*	13	13157011	13157526	177	20.28	8.09	Chloroplast
Pbr007600.1	*PbSNAT36*	14	406476	406937	159	18.15	8.03	Chloroplast
Pbr038809.1	*PbSNAT37*	14	16905552	16907791	215	24.42	6.20	Nucleus
Pbr019749.1	*PbSNAT38*	15	7194970	7197230	730	82.71	8.28	Nucleus
Pbr015568.1	*PbSNAT39*	15	15224166	15227132	457	50.91	5.41	Nucleus
Pbr023533.1	*PbSNAT40*	15	22526835	22528456	422	46.94	8.63	Chloroplast
Pbr024325.1	*PbSNAT41*	15	32914229	32919327	611	67.55	8.29	Chloroplast
Pbr008187.1	*PbSNAT42*	15	32914229	32919327	451	52.28	7.77	Nucleus
Pbr013684.1	*PbSNAT43*	16	9579818	9590057	1068	117.95	7.19	Cell membrane
Pbr022304.1	*PbSNAT44*	17	10688627	10691033	371	40.94	8.99	Nucleus
Pbr022307.1	*PbSNAT45*	17	10746962	10749368	371	40.94	8.99	Nucleus
Pbr021899.1	*PbSNAT46*	17	23875817	23876740	216	24.18	6.43	Chloroplast
Pbr005696.1	*PbSNAT47*	Scaffold1291.0	35688	41547	851	94.37	6.45	Nucleus
Pbr029344.1	*PbSNAT48*	Scaffold491.0	243198	245794	377	42.04	8.25	Nucleus
Pbr030260.1	*PbSNAT49*	Scaffold515.0	324717	325178	159	17.88	6.51	Chloroplast
Pbr034370.1	*PbSNAT50*	Scaffold640.0	69389	76084	1350	149.45	5.97	Nucleus
Pbr039064.1	*PbSNAT51*	Scaffold828.0	115475	117992	296	32.52	9.71	Chloroplast

Chr, Chromosome; AA, Amino Acid; MW, Molecular Weight; PI, Isoelectric Point; SL, Subcellular Location.

### 3.2 *PbSNAT* genes conserved domain analysis

**Figure 1:**
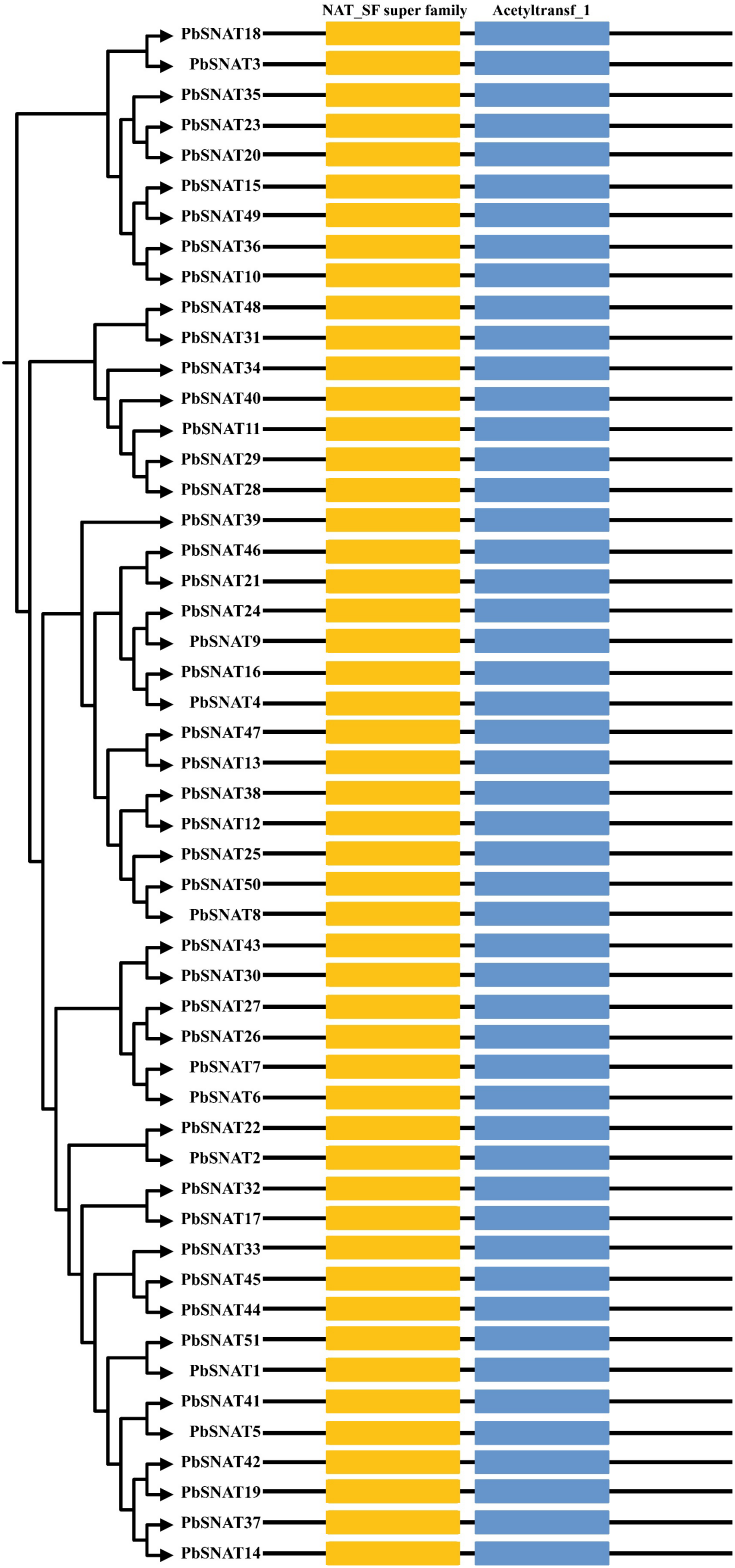
Schematic representation of the conserved domains of *PbSNAT* genes in *P. bretschneideri*.

Conserved domains within the PbSNAT proteins were identified using the Pfam and SMART databases. All PbSNAT proteins contained both the highly conserved NAT_SF superfamily and Acetyltransf_1 domain (**Figure 1**). These domains, ranging from 350 to 400 amino acids in length, span nearly the entire sequence of the PbSNAT proteins.

### 3.3 Chromosomal location of *PbSNAT* genes in *P. bretschneideri*

**Figure 2:**
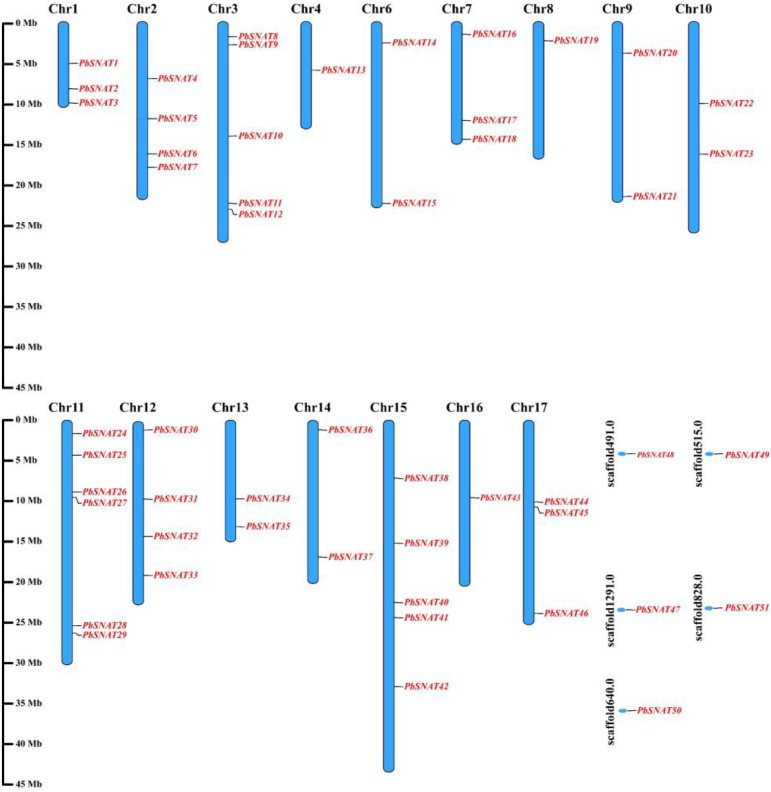
Chromosomal localization of *PbSNAT* genes in the *P. bretschneideri* genome. The relative positions of *PbSNAT* genes are marked on the chromosomes. The schematic representation was visualized using TBtools software.

The chromosomal locations of PbSNAT genes were mapped using the pear genome database and visualized with TBtools (**Figure 2**). The analyzed genes display a non-uniform distribution across the genome. A significant number of genes are localized on the main chromosomes (Chr1 to Chr17), while a subset is found on unplaced genomic scaffolds. On Chr3, two genes (*PbSNAT8*​ and *PbSNAT9*) are mapped in proximity, suggesting a potential tandem duplication event. Several chromosomes, including Chr2, Chr4, Chr6, Chr7, Chr8, Chr9, and Chr10, are shown to harbor only one or a few of the mapped genes in this analysis. The localization of five genes (*PbSNAT47*-*51*) on several scaffolds​ (scaffold491.0, scaffold515.0, scaffold526, scaffold527, scaffold530) indicates that portions of the genome containing these genes are not yet fully assembled into the pseudomolecules of the main chromosomes.

### 3.4 Evolutionary relationship of the *PbSNAT* gene family

**Figure 3:**
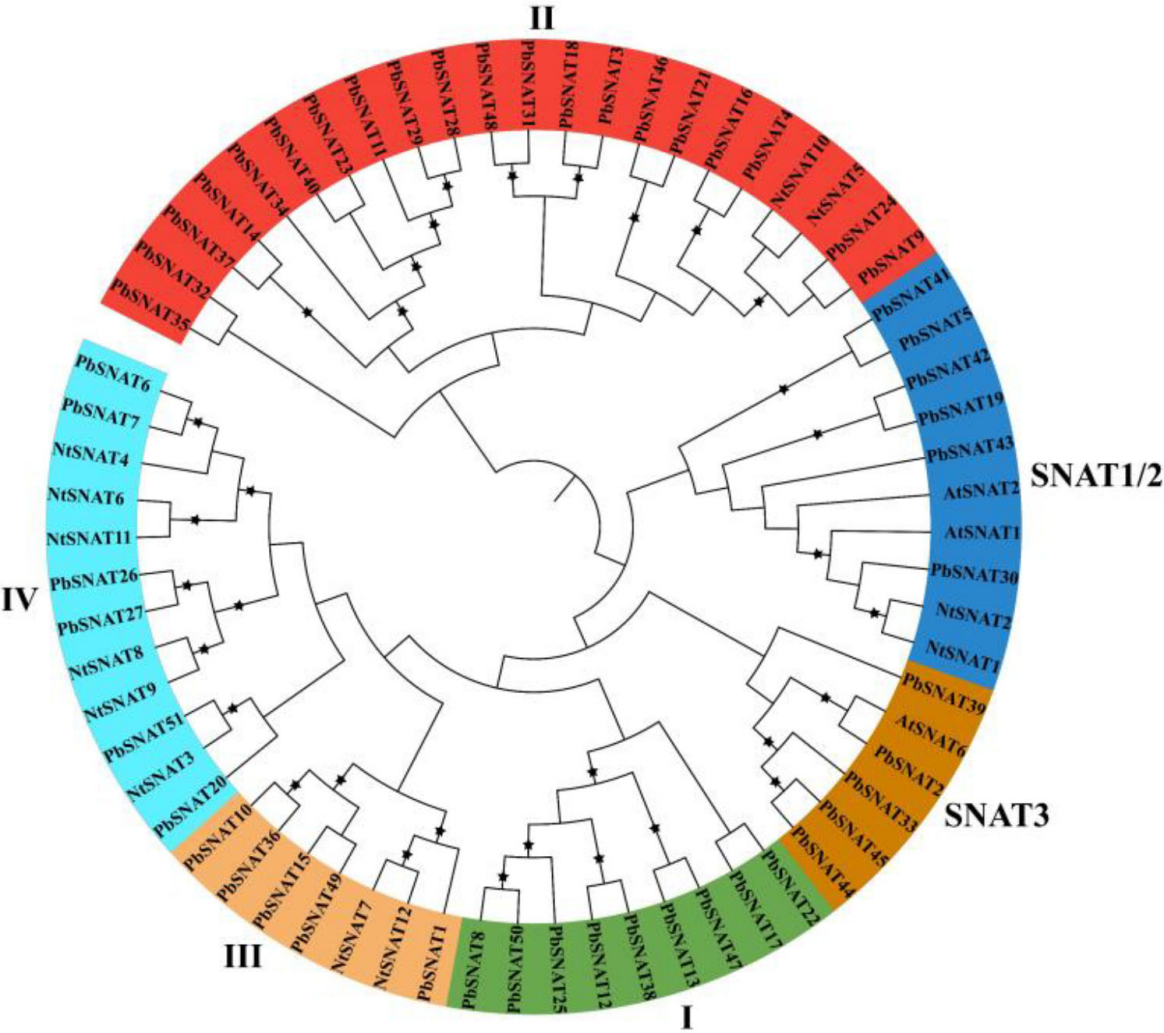
Phylogenetic analysis of SNAT family members across different plant species. The phylogenetic tree was generated using the amino acid sequences of selected SNAT via the Neighbor-joining tree method. All SNAT family members from Pear, *A. thaliana*, and *N. tabacum*, with their counterparts, were classified into six clusters, and the final tree was displayed using the Interactive Tree Of Life (iTOL) (version 5).

We employed Maximum Likelihood (ML) phylogenetic analysis to assess evolutionary relationships. The robustness of the inferred topology was evaluated with 1000 bootstrap replicates. Using amino acid sequences of pear, *A. thaliana*, and *N. tabacum* we classified the SNAT into six separate subfamilies (**Figure 3**). The co-occurrence of all three species proteins within each subfamily indicates a dynamic evolutionary history of gene gain and loss, which may underlie functional divergence in MT production. Accurately classifying these enzymes and resolving their evolutionary relationships establishes a critical foundation for elucidating their precise physiological roles in a species-specific context.

### 3.5 The *PbSNAT* genes structure analysis

**Figure 4:**
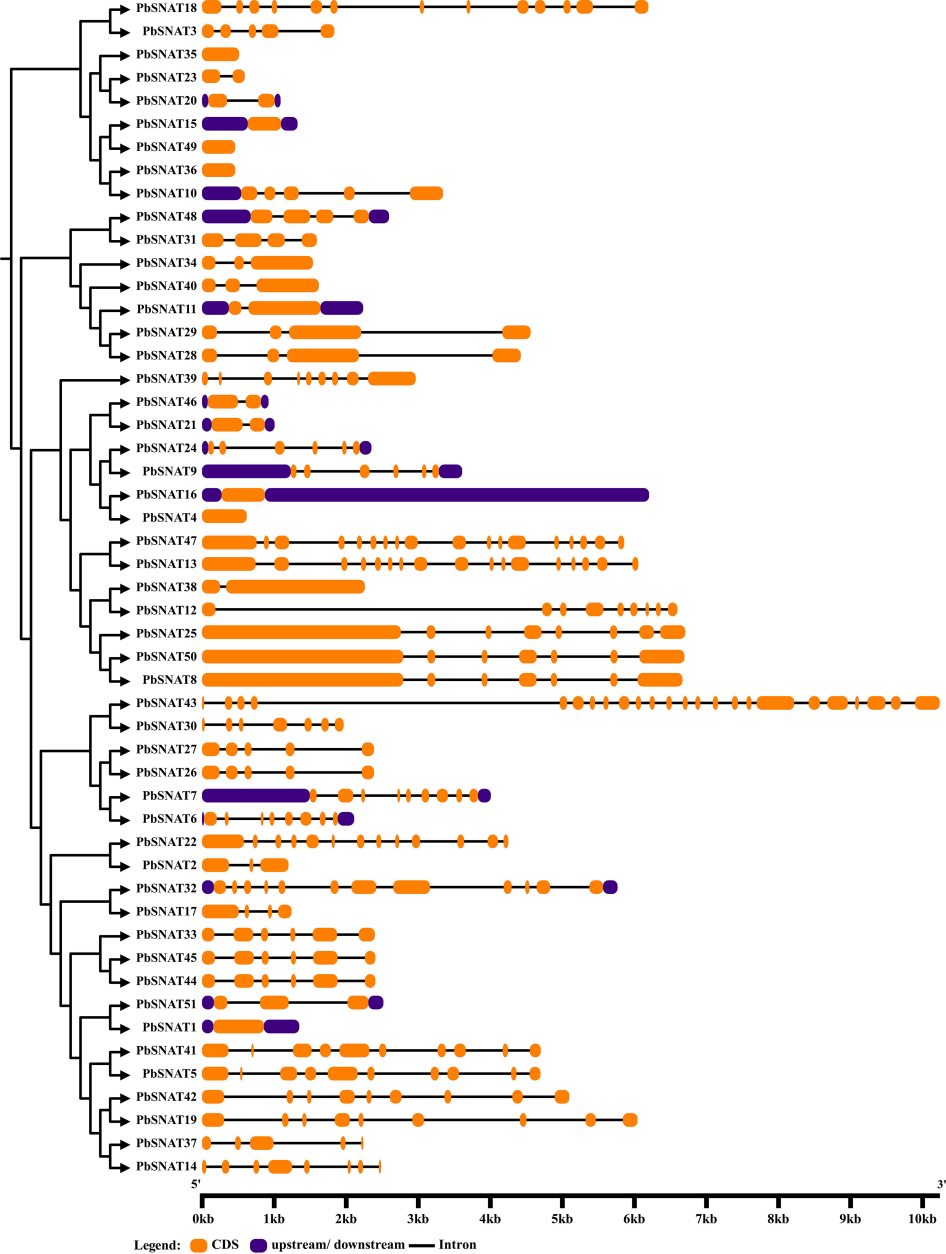
Locations and lengths of the exons and introns of *PbSNAT* family genes are depicted with exons as filled orange sticks, introns as thin black lines, and UTRs as bluish black bars. The gene structures were illustrated using the GSDS online database.

To analyze gene structure, we retrieved the complete coding sequences (CDS) and genomic sequences for the *PbSNAT* family from the pear genome database (**Figure 4**). The genes exhibit a range of exon counts, with most containing 4–6 exons. Notable exceptions include genes with fewer exons (*PbSNAT2/4/23/35/36/49*) or more exons (*PbSNAT43*), suggesting functional or regulatory divergence. Gene lengths span approximately 2–10 kb, primarily due to differences in intron sizes rather than exon number or length. Clusters of genes with highly similar exon-intron patterns (*PbSNAT3* and *PbSNAT10*) suggest recent gene duplication events​ with retained structural integrity. Furthermore, UTR regions (both upstream and downstream) were also identified in genes including *PbSNAT1/7/9/10* and several others (**Figure 4**).

### 3.6 Motif distribution of the *PbSNAT* genes in *P. bretschneideri*

**Figure 5:**
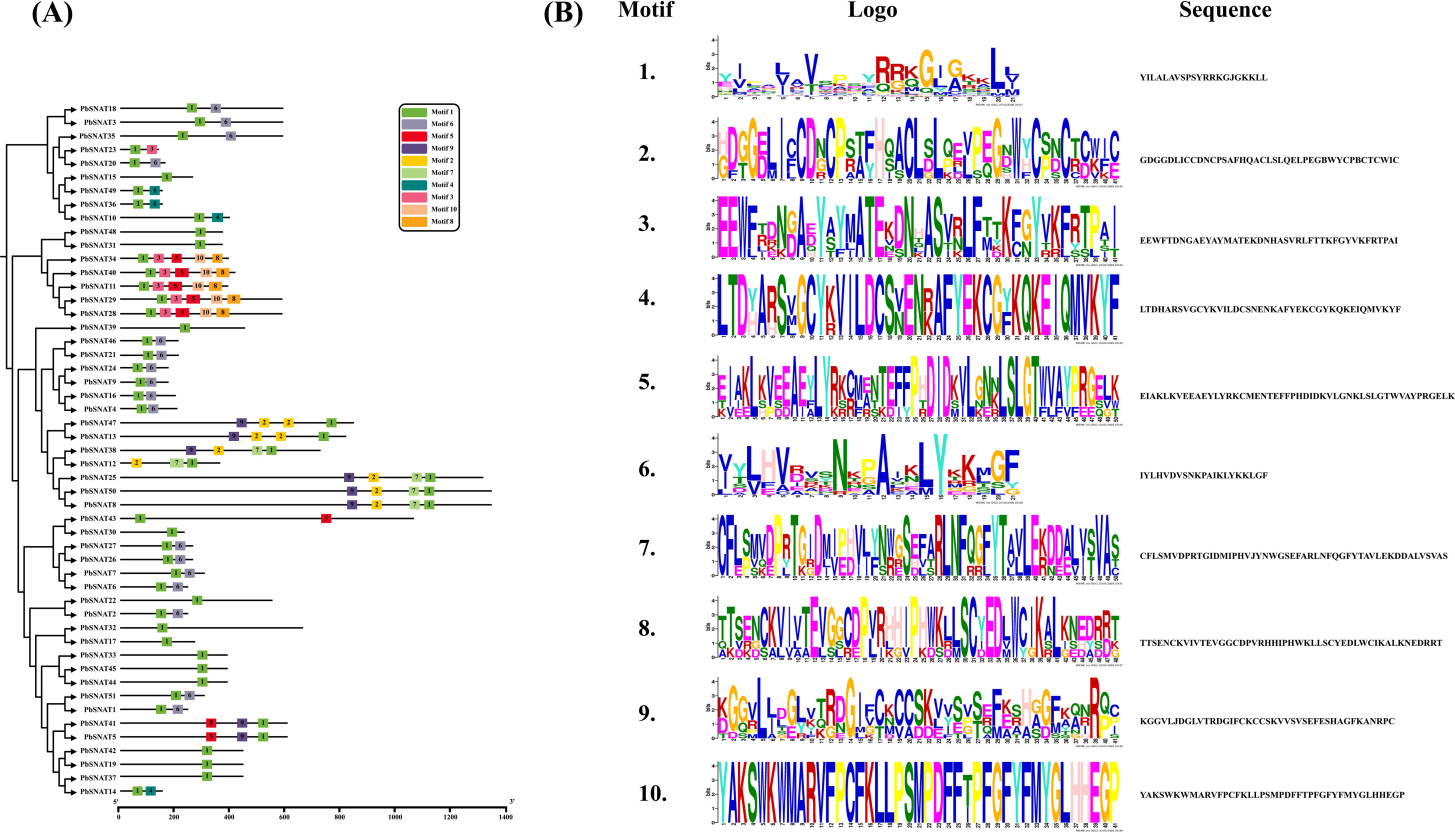
Schematic representation of the conserved motifs of *PbSNAT* **(A)** and their corresponding sequences **(B)** in *P. bretschneideri*.

MEME analysis (*E-value > 1.2 × 10−221*) identified ten conserved motifs in the amino acid sequence of PbSNAT genes (**Figure 5**). Motif 1 was detected in all the PbSNAT proteins, showing its high conservancy. The Motif 10 was detected in PbSNAT11, PbSNAT28, PbSNAT29, PbSNAT34, and PbSNAT40, but was not found in the remaining PbSNAT proteins. Similarly, motif 8 was noted in PbSNAT11, PbSNAT28, PbSNAT29, PbSNAT34, and PbSNAT40, but was not detected in other members of the identified pear SNAT proteins (**Figure 5**).

### 3.7 *C*is regulatory elements of *PbSNAT* genes in *P. bretschneideri*

**Figure 6:**
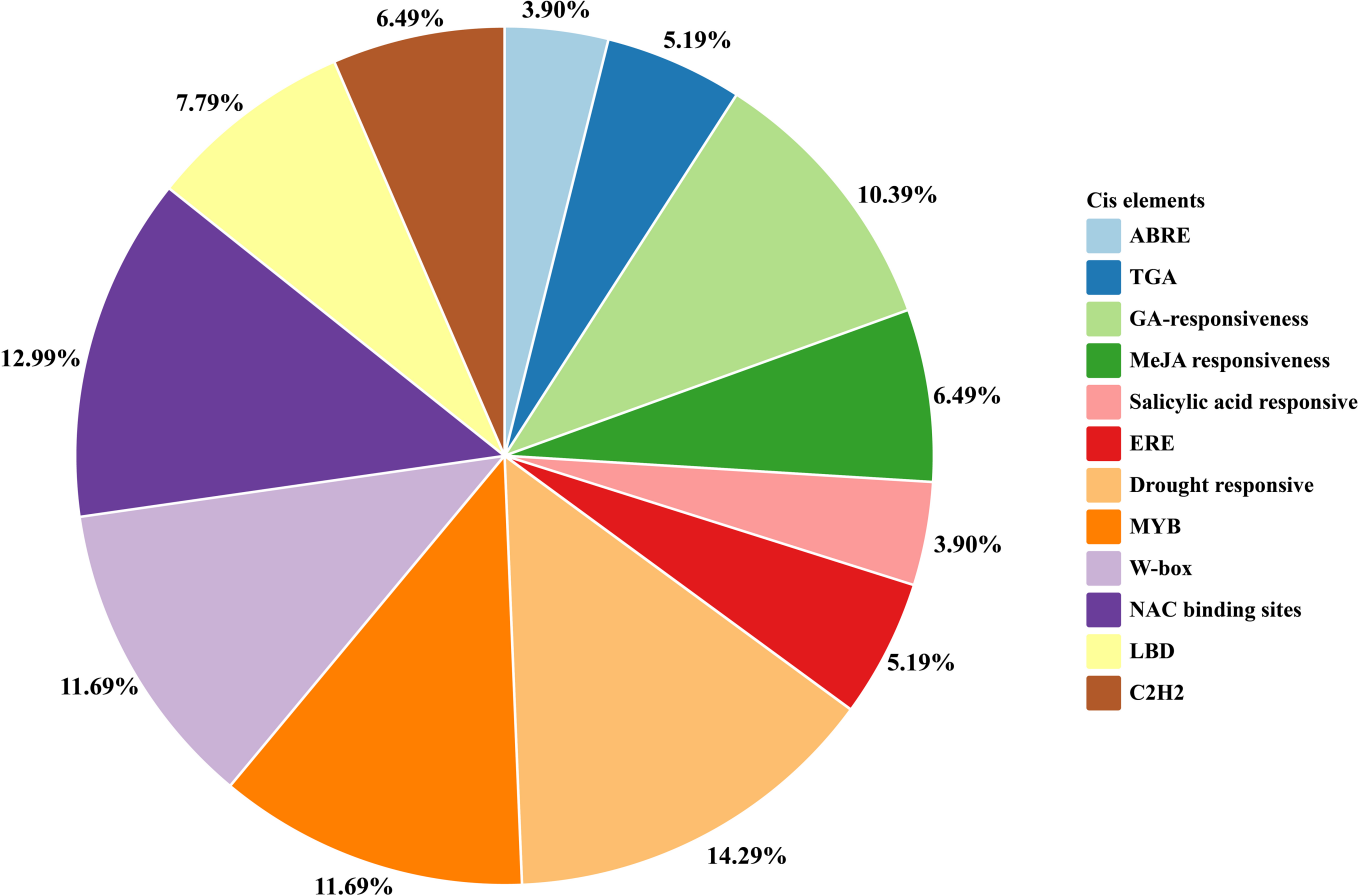
*Cis*-acting elements are presented in the 1.5-kb upstream region of *PbSNAT* genes. Various categories of *cis*-elements were retrieved, and the image was drawn on GraphPad Prism software.

To investigate transcriptional regulation, we analyzed the 1.5 kb promoter region upstream of the ATG start codon for all *PbSNAT* genes using the PlantCARE database (**Figure 6**). The analysis identified numerous *cis*-elements associated with hormone signaling, stress responses, and development. For instance, the promoter of *PbSNATs* was enriched for hormone-responsive elements, followed by stress-related ones. Prominent hormone-related *cis*-elements included 3.90% ABRE (ABA-responsive element), 5.19%TGA (auxin-responsive cis-acting element), 10.39% GA-responsiveness, 6.49% MeJA (Methyl jasmonate) and 3.90% salicylic acid. Key stress-responsive elements, such as NAC, W-box, MYB, ERE, and drought-responsive motifs, were also prevalent across the *PbSNATs* promoters (**Figure 6**). A high percentage of NAC (12.99%) binding sites were the most prominent.

### 3.8 Synteny analysis of *PbSNAT* family members

**Figure 7:**
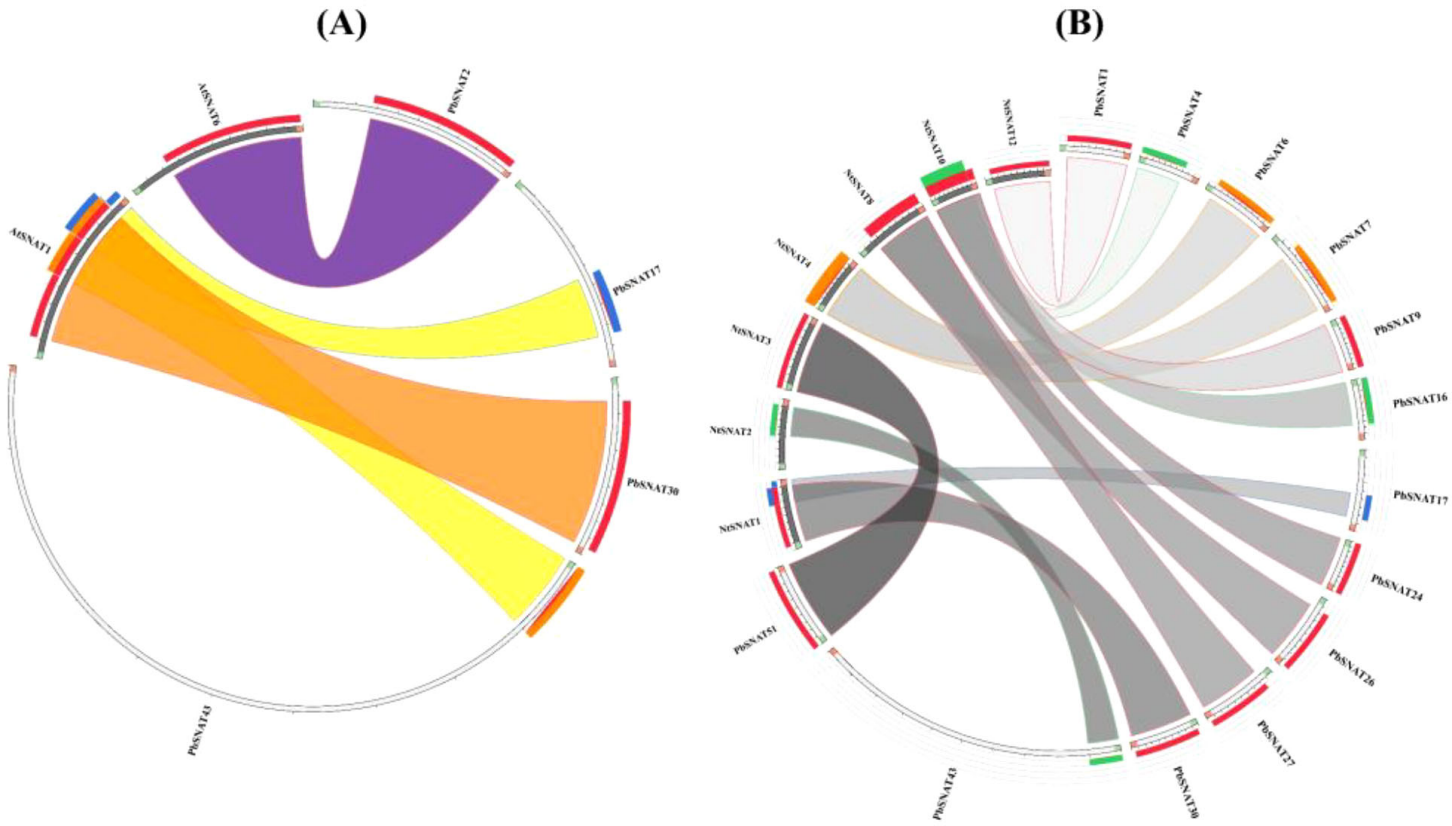
Synteny analysis of SNAT genes between **(A)** Pear and *A. thaliana* and **(B)** Pear and *N. tabacum*. The chromosomes of Pear, *Arabidopsis*, and *N. tabacum* are arranged as a circle. Colored lines represent syntenic occurrences of *PbSNAT* genes.

To understand the evolutionary history and potential functional diversification of *PbSNAT* genes, we analyzed gene duplication events and examined synteny between pear, tobacco, and Arabidopsis. Comparative genomic analysis revealed extensive synteny, with approximately 40% of Arabidopsis and 56% of pear SNAT genes residing in conserved collinear blocks (**Figure 7A**). Comparative analysis also revealed strong collinearity, with approximately 62% of pear genes and 40% of tobacco (*N. tabacum*) genes residing in conserved syntenic blocks (**Figure 7B**). These widespread syntenic relationships highlight both the shared evolutionary origins and the significant chromosomal rearrangements that have occurred since the divergence of these species.

### 3.9 Gene ontology analysis of *PbSNAT* family members

**Figure 8:**
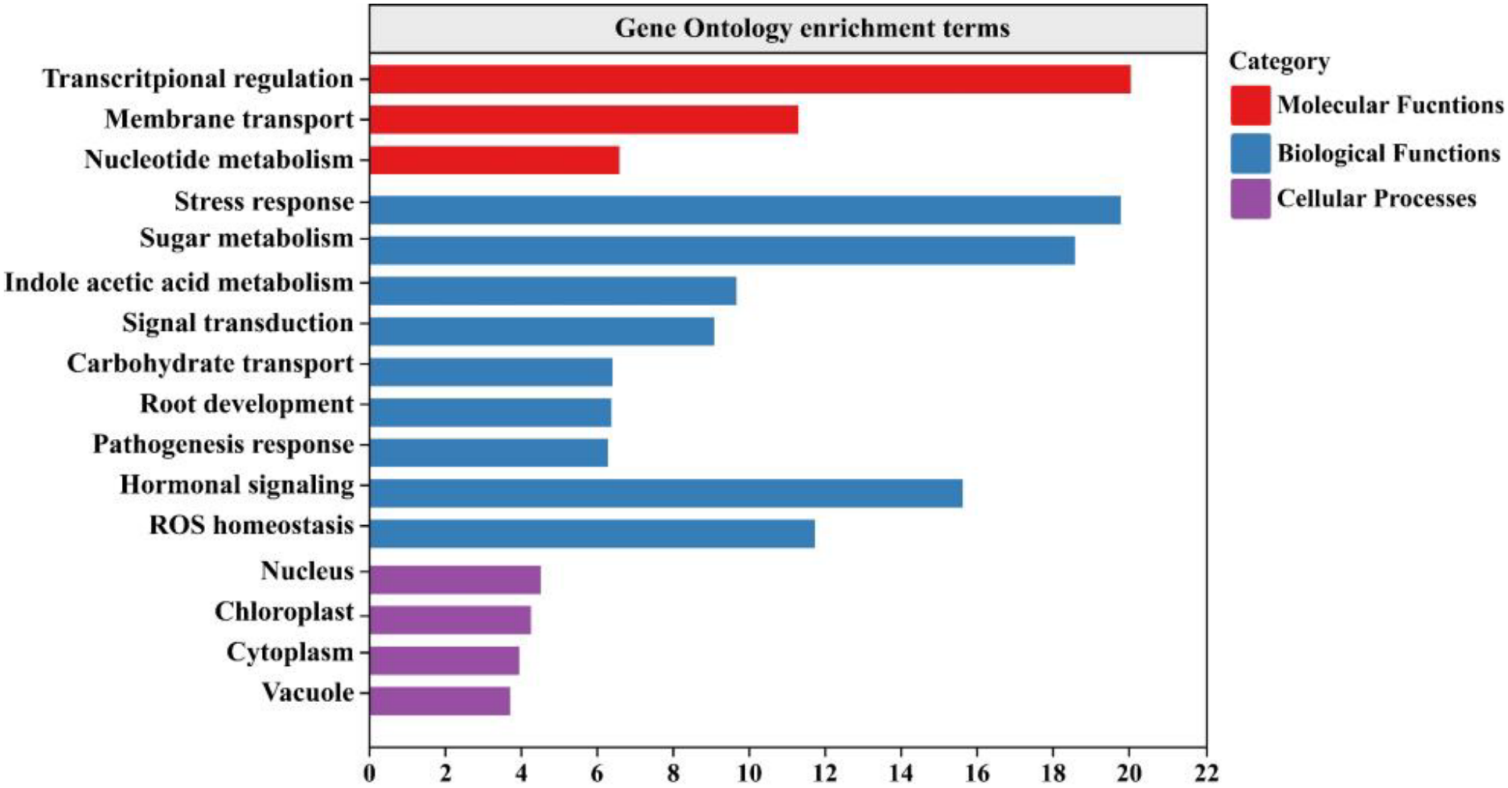
Gene ontology analysis of *PbSNAT* genes shows the distribution of various biological, molecular, and cellular processes.

To predict the potential functions of the *PbSNAT* genes family, we performed a gene ontology (GO) enrichment analysis using the encoded protein sequences (**Figure 8**). The results confirmed a significant role in indole acetic acid metabolism. Furthermore, the most enriched GO terms revealed critical involvements in hormonal regulation and sugar metabolism, distinguishing the functional profile of *PbSNAT* proteins. This enrichment pattern positions the *PbSNAT* family at a key intersection of metabolic and signaling networks. The link to hormone regulation suggests a mechanism for MT to interact with other phytohormone pathways, while the association with sugar metabolism points to a possible role in integrating metabolic status with stress-responsive signaling, a function crucial for perennial species like pear.

### 3.10 Interactive proteins network analysis

**Figure 9:**
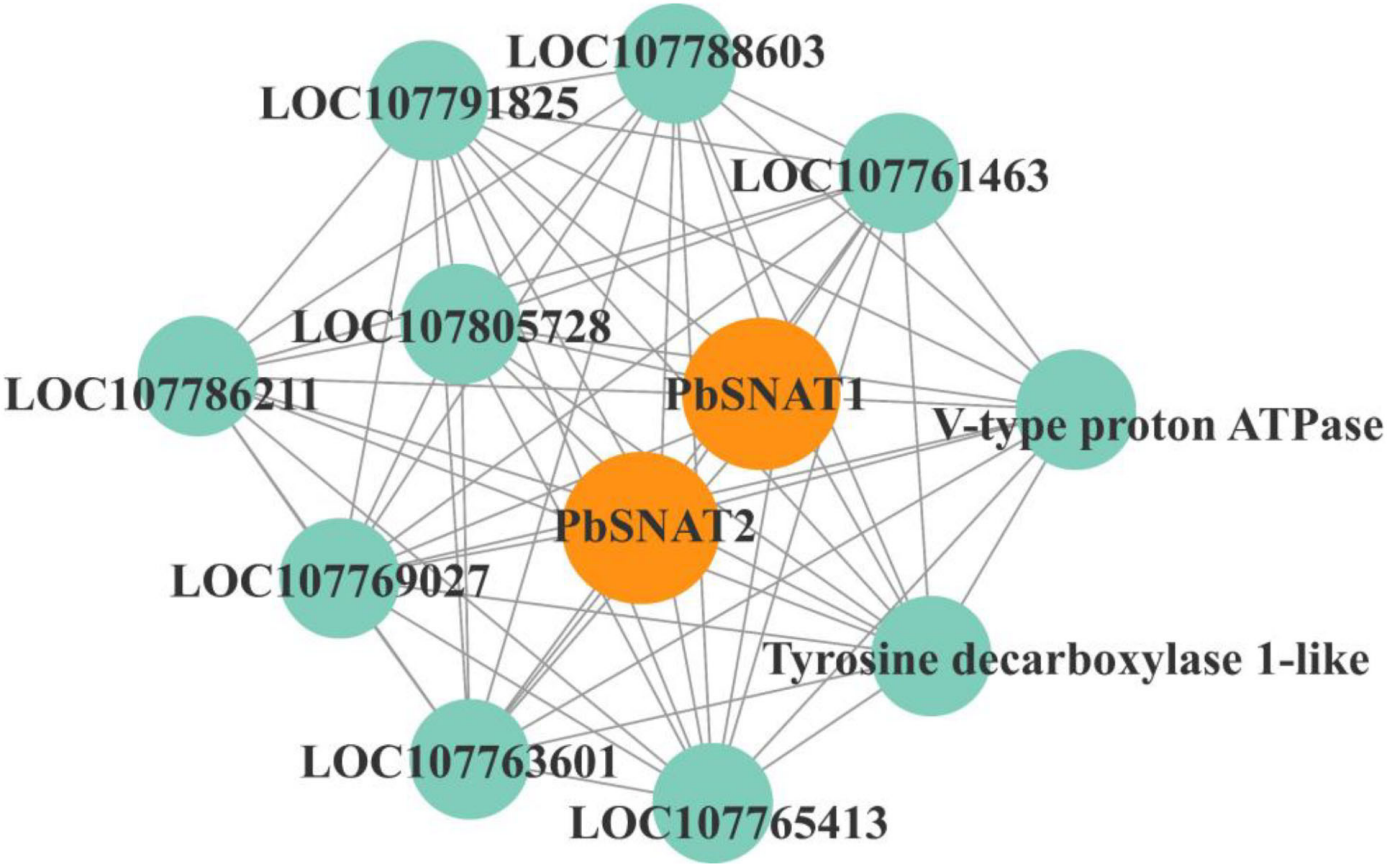
Interactive protein network of PbSNAT. All the PbSNAT proteins were used as references, which displayed interaction with numerous other key proteins.

Analysis via the string database revealed a potential interaction network for PbSNAT proteins (**Figure 9**). *PbSNAT1* emerged as a highly connected hub. A significant interaction partner was V-type proton ATPase (V-ATPase), the key enzyme regulating cytosolic pH and providing energy for vesicular transport. This interaction suggests a possible link between MT biosynthesis and intracellular pH homeostasis or membrane trafficking. Localization in the trans-Golgi network/early endosomes is crucial for vesicle transport, particularly for the delivery of cell wall components. Tyrosine decarboxylase (TyDC) is an enzyme that relies on pyridoxal-5′-phosphate (PLP) to catalyze the decarboxylation of L-tyrosine (Tyr), resulting in the production of the biologically significant tyramine.

### 3.11 Protein 3D model analysis

**Figure 10:**
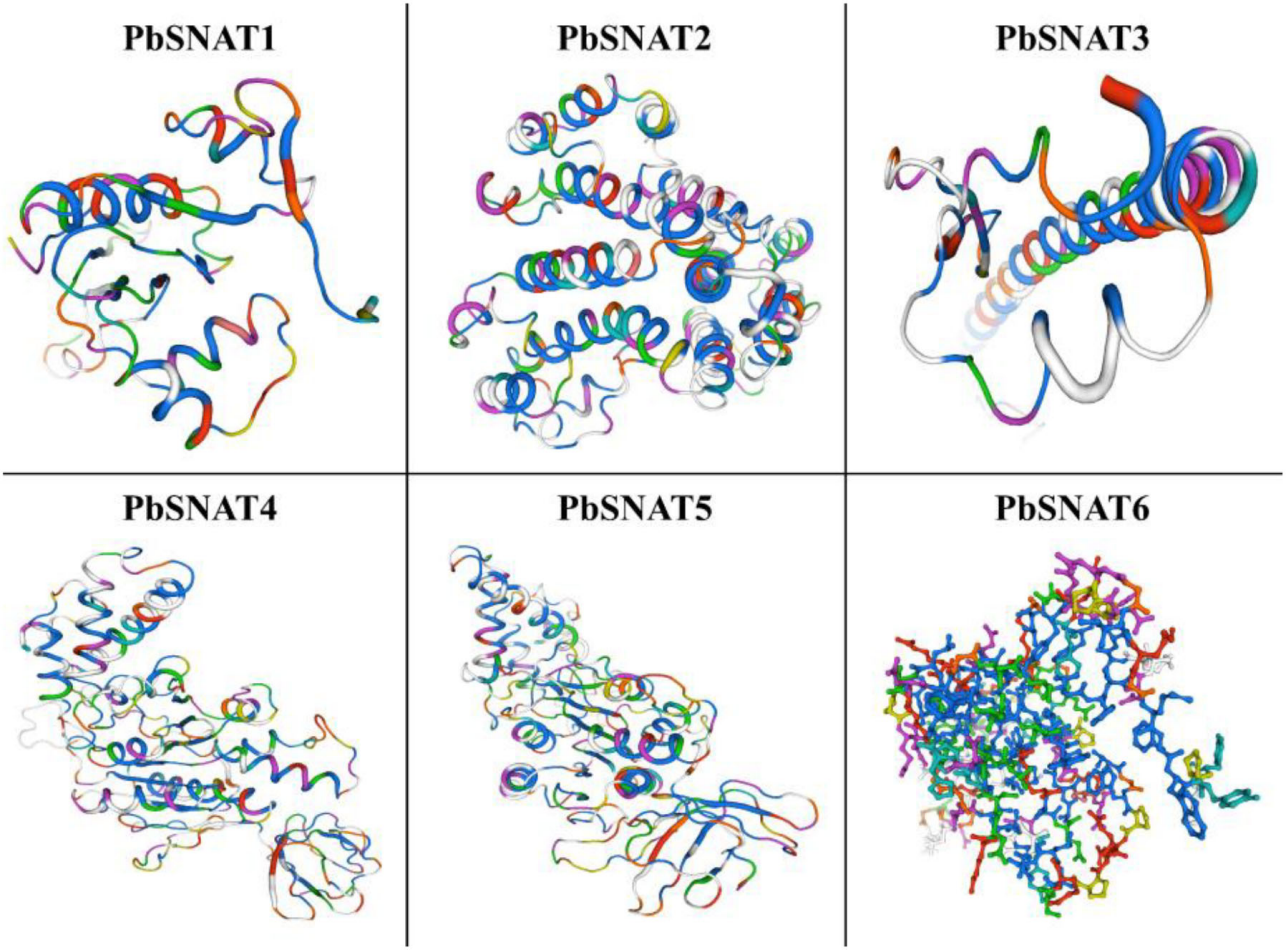
The 3D protein homology of PbSNAT1-PbSNAT6. The Expasy server was used to draw the 3D protein structure. Protein-protein docking and hydrogen bond interactions observed in the binding interface region of PbSNAT1-PbSNAT6 by the molecular docking technique.

The three-dimensional structures of PbSNAT proteins were predicted with an overall confidence score of 76%, and their potential active sites were identified. All predicted models exhibited a conserved architecture characterized by a distinctive fold of several parallel β-strands and α-helices. To comprehend their structural features, we have supplied several PbSNAT 3D models (**Figure 10**). These results point to a shared structural characteristic among these proteins. The PbSNAT proteins exhibit significant similarity, especially in their catalytic sites and regions responsible for binding metal ions, despite minor variations in their sequences (**Figure 10**). The results of the ProSA (Protein Structure Analysis) confirmed that the modeling quality was present in various areas of the proteins, as each model contained regions with a high percentage of the lowest energy residues. PbSNAT1 and PbSNAT4 show more compact folds, which may correlate with higher stability. In contrast, PbSNAT5 appears more extended, suggesting flexible active sites or interaction interfaces.

### 3.12 Predicted miRNAs and network analysis

**Figure 11:**
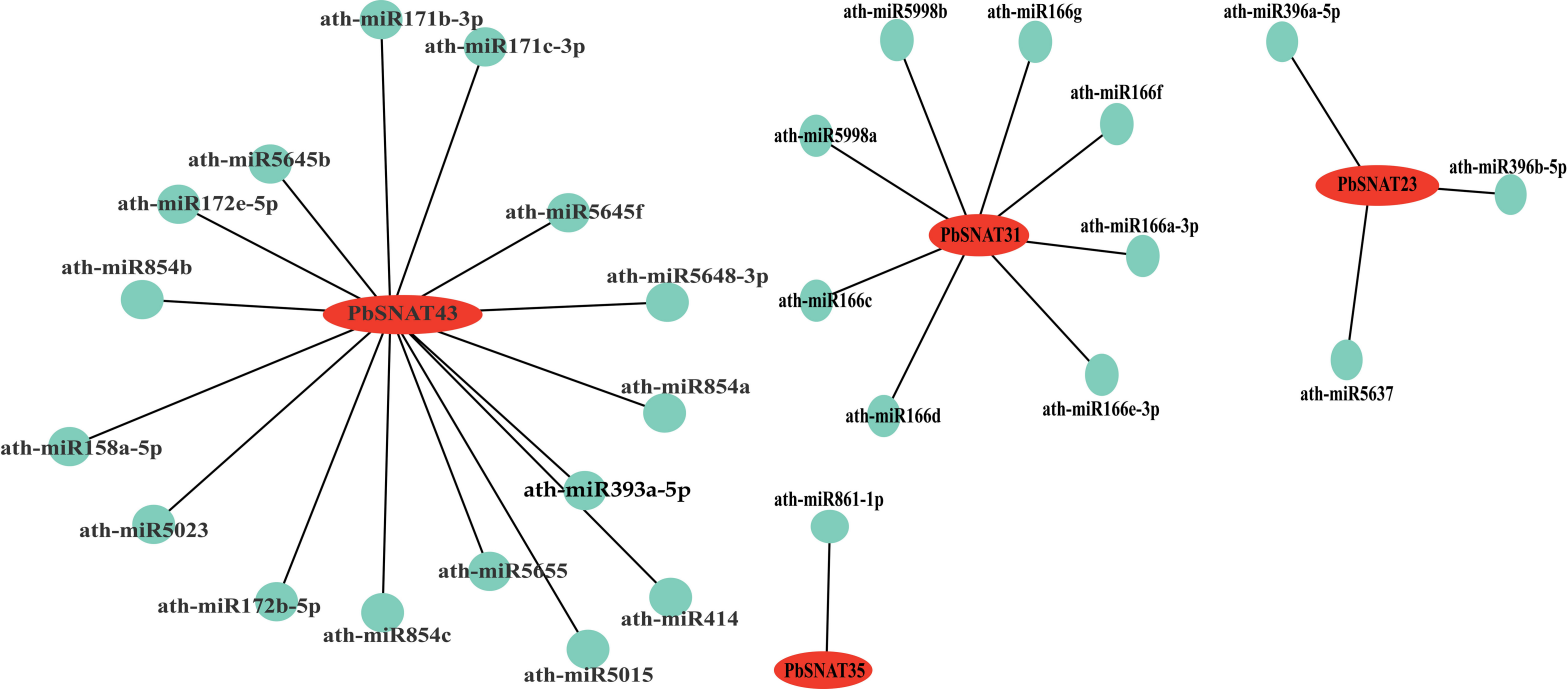
Predicted targeted miRNAs cleaved by *PbSNAT* genes. The Cytoscape was used to draw the miRNA-gene network. The *PbSNAT* genes are represented with red color, whereas the and green circles represent targeted miRNAs.

The downloaded sequences of all pear miRNAs were compared with the coding sequences (CDSs) of *PbSNAT* genes to predict miRNAs potentially targeting and cleaving these genes. The miRNA library we have consists of many PbSNAT genes, such as *PbSNAT23*, *PbSNAT31*, *PbSNAT35*, *PbSNAT43*, and several others (**Figure 11**). The *PbSNAT23* cleaved to a well-known miRNA families, including miR396 and miR5637. Additional important miRNA families comprising miR166, miR171, miR172, miR393, and miR854 are noticed (**Figure 11**). These miRNAs are primarily responsible for driving a significant portion of biological activities in plants.

### 3.13 Tissue-specific expression analysis of *PbSNAT* genes in *P. bretschneideri*

**Figure 12:**
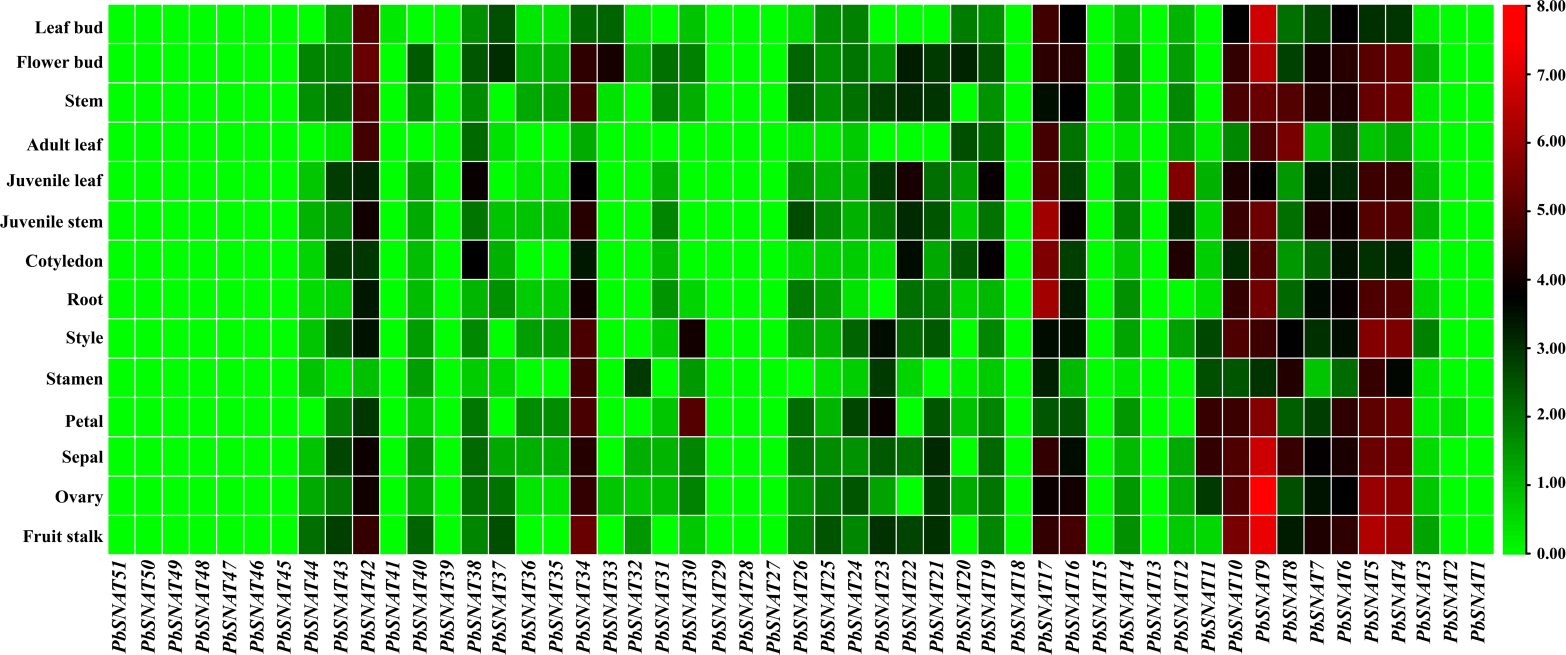
Heatmap of *PbSNAT* gene family transcript expression profiles from *P. bretschneideri* RNA-seq dataset. Colored boxes in each column show gene expression relative to log_2_ (Fold change). Data was obtained from the stem, fruits, ovary, leaf, bud, petal, and sepal. Levels of expression are displayed as red for higher and green for lower. The TBtools software was used to create the heatmap.

We analyzed the expression of *PbSNAT* genes in different organs of *P. bretschneideri* using RNA-seq data (**Figure 12**). Expression profiling of the *PbSNAT* gene family revealed pronounced tissue-specific patterns, with a clear distinction between vegetative and reproductive tissues (**Figure 12**). While most genes showed low expression in vegetative organs (stem, leaf bud, leaf), *PbSNAT9* was a notable exception, exhibiting consistently high expression across all tissues. In reproductive organs (petals, sepals, ovary, fruit), expression was more dynamic. A subset of genes, including *PbSNAT3*, *PbSNAT7*, *PbSNAT9*, and *PbSNAT10*, was highly expressed in floral tissues. During fruit development, this pattern shifted as *PbSNAT9* maintained high expression, whereas *PbSNAT3* and *PbSNAT7* expression declined. Distinctly, *PbSNAT6* and *PbSNAT13* showed elevated expression specific to fruit tissues (**Figure 12**).

### 3.14 Expression analysis *PbSNAT* genes in five different species and quantification of endogenous MT and SNAT

**Figure 13:**
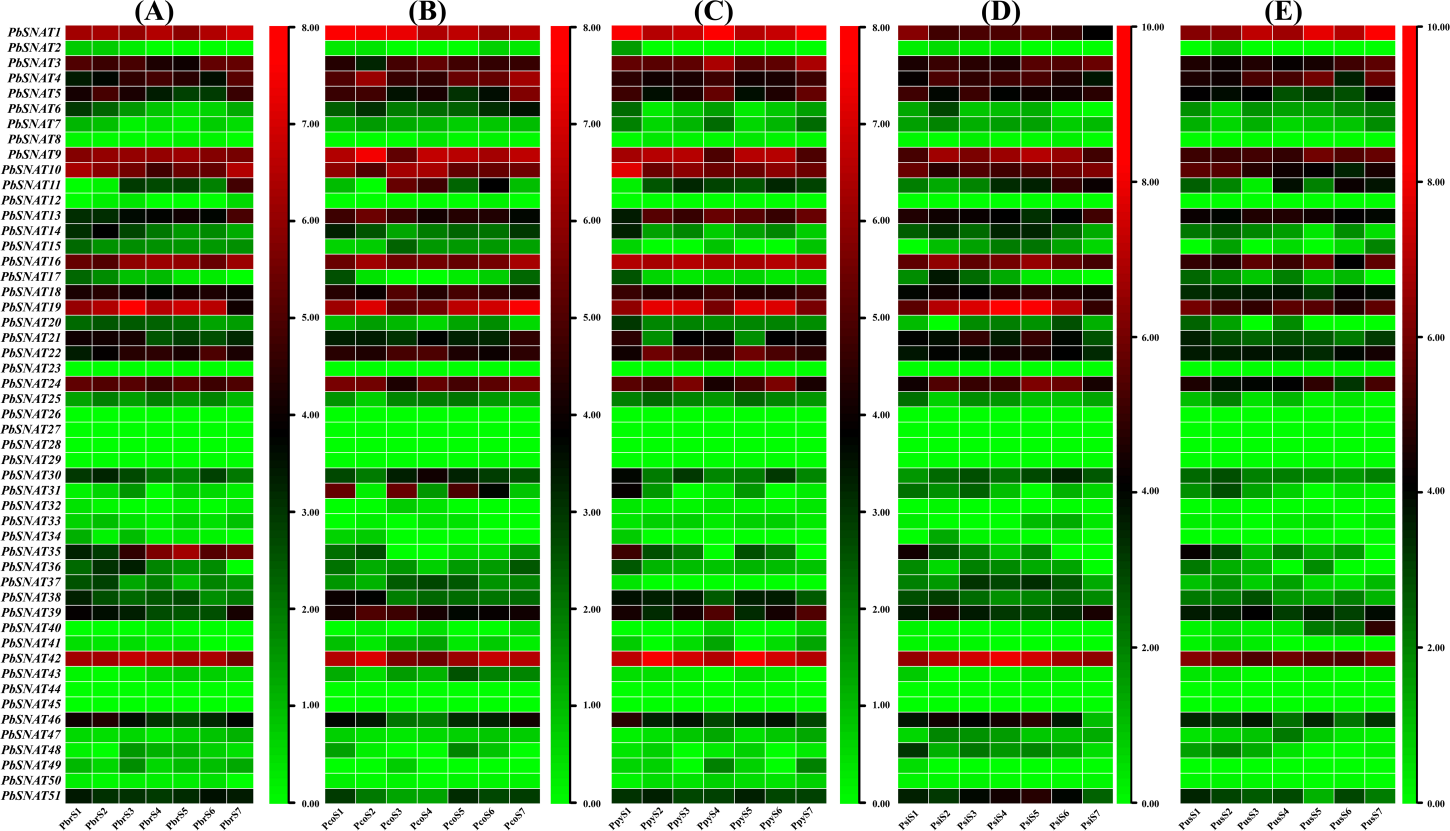
A heatmap depicting the transcript expression profiles of the *PbSNAT* gene family across various RNA-seq datasets from different pear species. **(A)** *P. bretschneideri* (Pbr), **(B)** *P. communis* (Pco), **(C)** *P. pyrifolia* (Ppy), **(D)** *P. sinkiangensis* (Psi), and **(E)** *P. ussuriensis* (Pus). Tbtools software generated the heatmap. The red boxes indicate increased gene expression, while the green boxes represent decreased gene expression.

**Figure 14:**
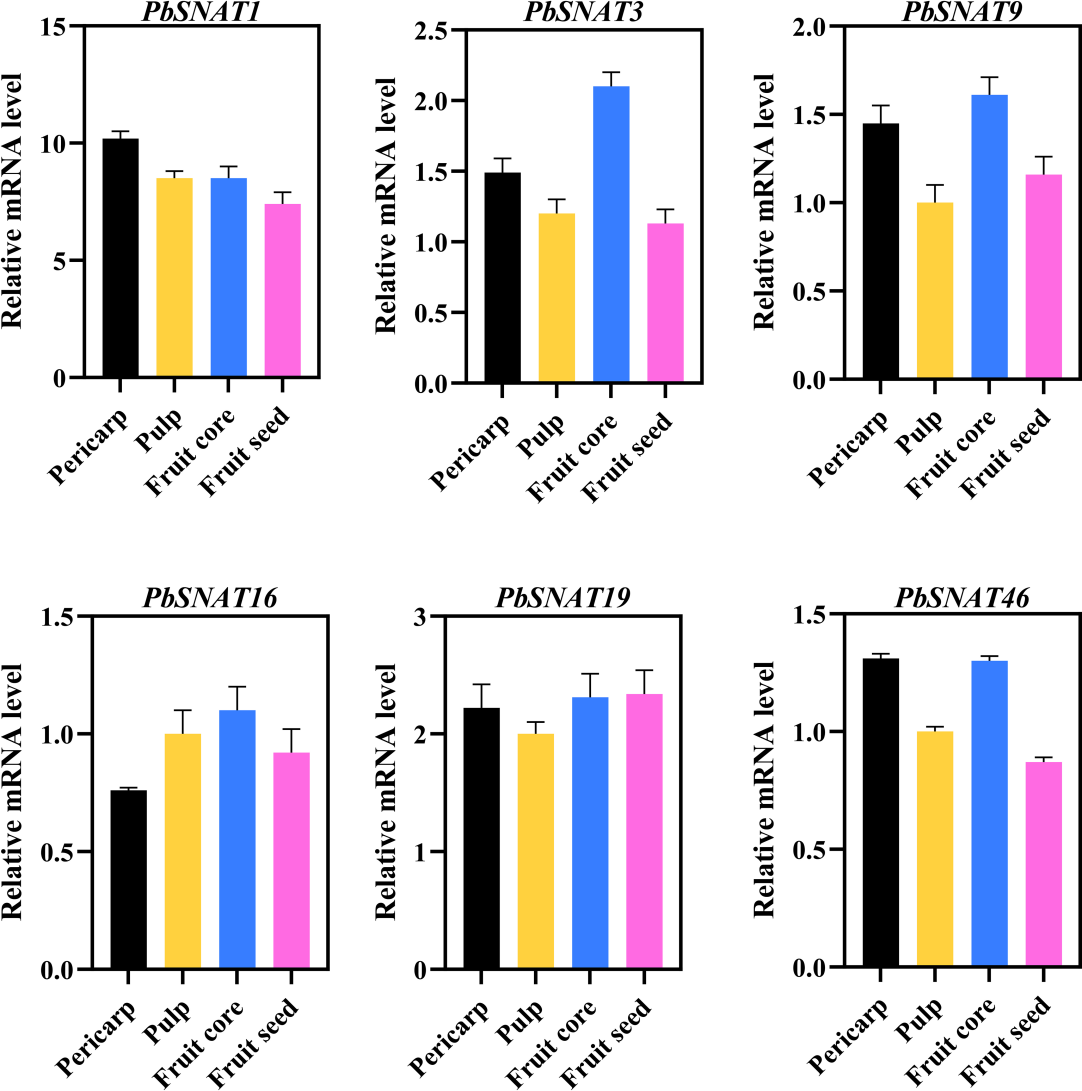
Tissue-specific expression of *PbSNAT* genes in pear fruit tissues. Expression was analyzed in fruit pericarp (black), pulp (gold), core (blue), and seed (pink) tissues. Values represent normalized relative mRNA levels (mean ± SE).

This study included five distinct pear cultivars: Pbr (*P. bretschneideri*), Pco (*P. communis*), Ppy (*P. pyrifolia*), Psi (*P. sinkiangensis*), and Pus (*P. ussuriensis*) (**Figures 13A–E**). To further investigate *PbSNAT* gene expression, we analyzed samples collected at seven developmental stages (S1–S7) during fruit growth. While multiple genes showed elevated expression, *PbSNAT1* was particularly prominent, exhibiting a substantial and progressive increase in mRNA levels from S1 to S7 across all five pear cultivars (Pbr, Pco, Ppy, Psi, and Pus) examined (**Figure 13**). Expression patterns for the majority of other *PbSNAT* genes were largely conserved among these species, suggesting a core conserved function. To further confirm these tissue-specific expression patterns, we performed qRT-PCR analysis. The results revealed distinct expression profiles for each *PbSNAT* gene across different Pear species transcriptomic datasets. For instance, *PbSNAT1* expression was highest in the pericarp, *PbSNAT3* peaked in the fruit core, and *PbSNAT9* showed high expression in both tissues (**Figure 14**). This confirms the primary fruit tissue in which each gene functions.

**Figure 15:**
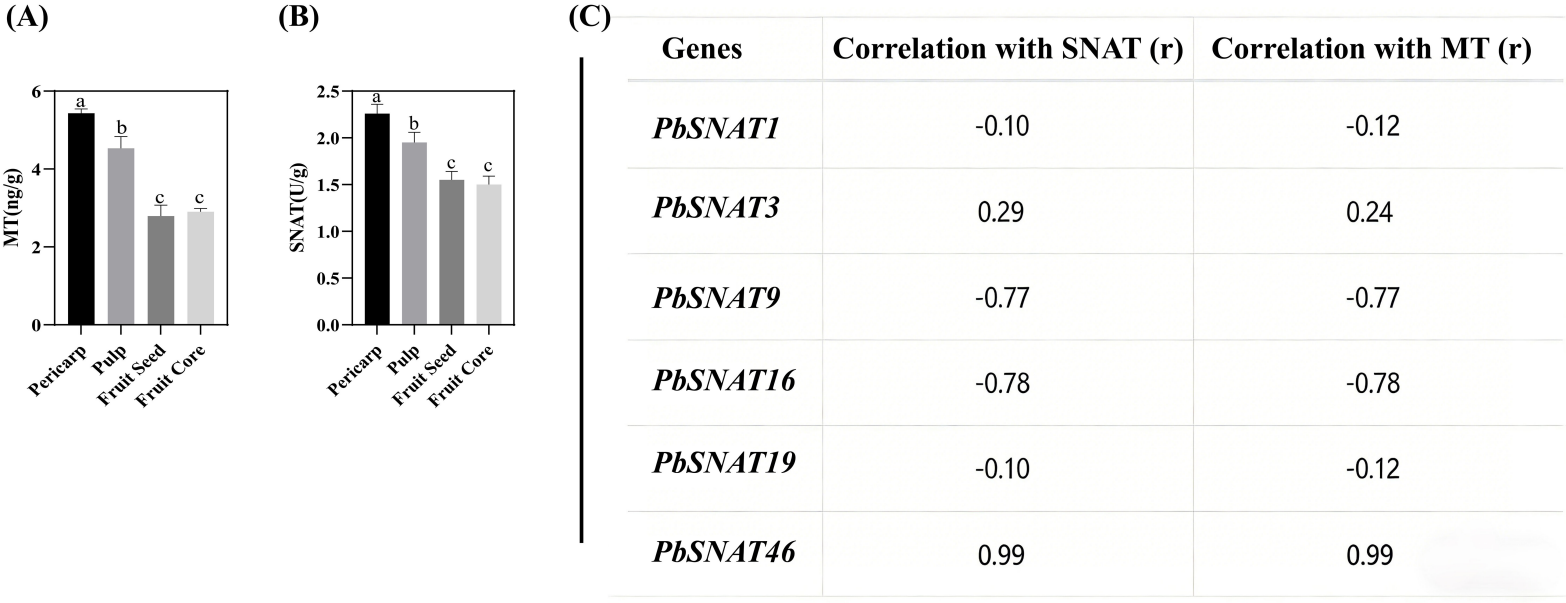
Tissue-specific accumulation of MT and SNAT, and their correlation with *PbSNAT* gene expression in pear fruit. **(A)** MT content in different fruit tissues (pericarp, pulp, fruit seed, and core). Data are presented as mean ± SD (n=3). Different lowercase letters above the bars indicate statistically significant differences among tissues (*p* < 0.05). **(B)** SNAT content in different fruit tissues. Data are presented as mean ± SD (n=3). Different lowercase letters above the bars indicate statistically significant differences among tissues (*p* < 0.05). **(C)** Pearson correlation coefficients (r) between the expression levels of six *PbSNAT* genes and the contents of SNAT and MT across the tissues studied.

The analysis of MT content and SNAT enzyme activity across four distinct fruit tissues revealed a clear and consistent tissue-specific pattern (**Figures 15A–C**). The distribution of MT content and SNAT activity across different fruit tissues was significantly different. In the fruit pericarp, both MT concentration and SNAT activity reached their highest levels, measuring 6 ng/g and 2.5 U/g, respectively. These values were significantly greater than those found in all other tissues examined. The pulp exhibited intermediate levels for both parameters, with an MT content of 4 ng/g and SNAT activity of 2.0 U/g. These values were significantly lower than those in the pericarp but significantly higher than those in the seed and core. The fruit seed and fruit core showed the lowest and statistically similar levels of MT, both at 2 ng/g. For SNAT activity, the seed and core also demonstrated the lowest activities, measuring 1.5 U/g and 1.0 U/g, respectively. The activity in both tissues was significantly lower than that in the pericarp and pulp (**Figures 15A, B**). The mRNA expression pattern of *PbSNAT46* shows an exceptionally strong (**Figure 15C**), near-perfect positive correlation with both SNAT activity (r = 0.99)​ and MT content (r = 0.99). This quantitatively confirms the visual observation that its expression is highest in the pericarp, high in pulp, and low in seed and core, exactly mirroring the patterns of enzyme activity and final product concentration.

### 3.15 Electronic fluorescent pictographic (eFP)of *PbSNAT1* in pear tissues

**Figure 16:**
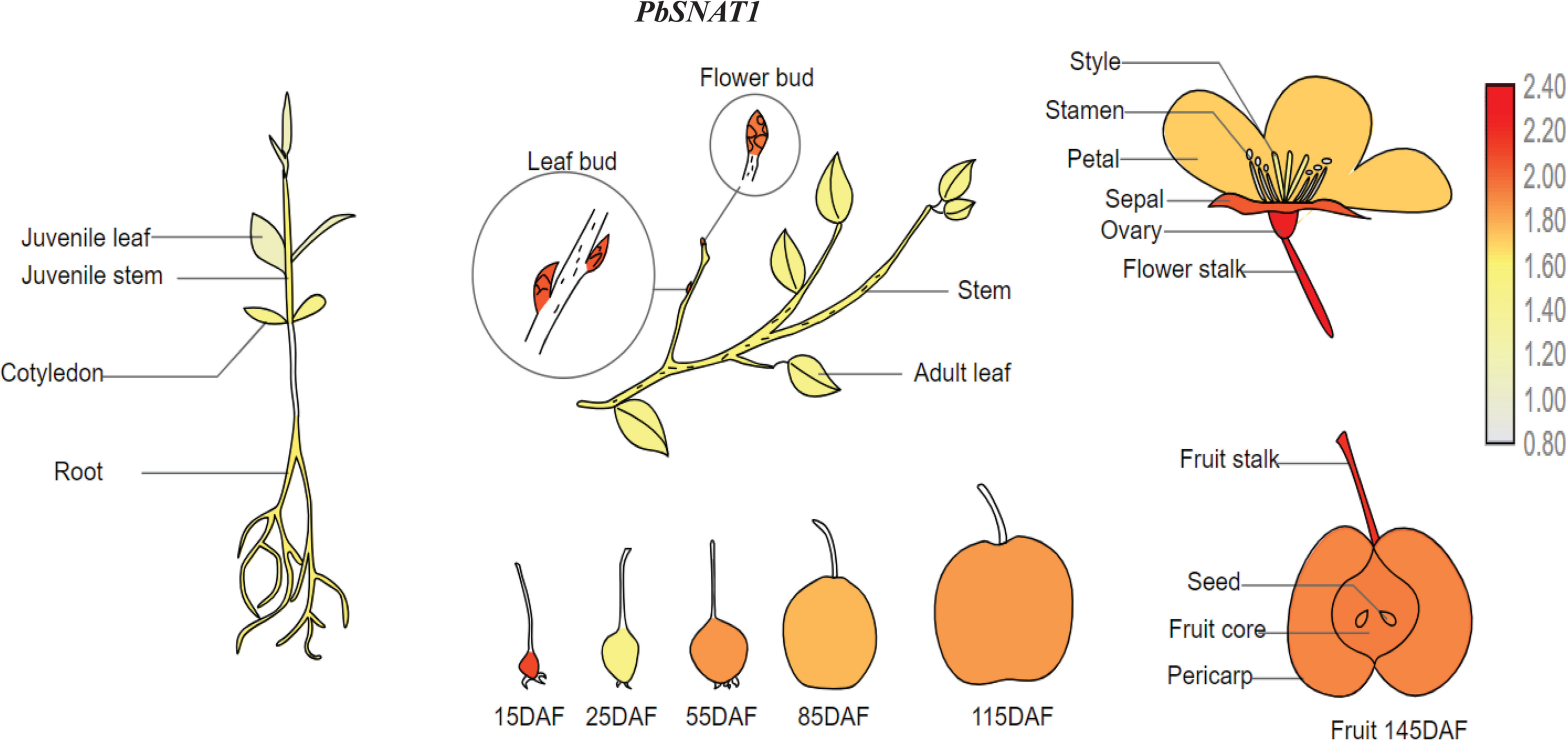
A schematic showing the distribution of *PbSNAT1* expression in various pear tissues. Various organ names are shown. The white-grey indicates modest expression, while the dark red indicates high expression.

Based on its high expression across all five-pear species, we provide a visual depiction of *PbSNAT1* expression in pear tissues (**Figure 16**). As shown in the model, the *PbSNAT1* gene showed significant expression levels throughout the pear life cycle. Given this, *PbSNAT1* could be utilized as a marker for investigating its role in fruit development and maturity.

## 4 Discussion

Members of the SNAT family, which is a subfamily of the GNAT superfamily, are essential regulators of MT accumulation (Lee et al., 2015). SNAT genes have been detected in diverse plant species (Yu et al., 2019). One useful method for identifying genetic variations across an organism genome is a genome-wide association analysis (Yang et al., 2021). In this work, we performed extensive bioinformatics and expression investigations on the pear genome and found 51 *PbSNAT* genes.

### 4.1 SNAT genes are widely distributed in the pear genome

These genes were split into six branches by the evolutionary tree, and the SNAT gene distribution was found in each branch (**Figure 3**). Based on the genomic distribution of *SNAT* genes, it was found that both dicotyledons and monocotyledons had *SNAT* genes in their ancestral genomes. This further proves that prior to the split between dicots and monocots, the *SNAT* gene family had already formed clades I, II, III, IV, V, and VI. Clade I has the highest number of *SNATs* in nearly all plants, suggesting that it may be an old group of *SNAT* genes. This indicates the significant role and contribution of clade I in the extensive growth of higher plants (Malik et al., 2020). According to previous reports, *SNAT* is found in chloroplasts, which aligns with our predictions (Byeon et al., 2014). Further, the investigation of gene structure shows that four genes lacked introns (**Figure 4**). Genes with fewer introns can rapidly evolve through replication or reverse transcription and then become integrated into the genome (Lurin et al., 2004). The evolution of novel functions in gene families with reduced introns is believed to occur through a mechanism involving replication or reverse transcription, followed by integration into the genome (Lurin et al., 2004). A gene motif is a brief sequence of characteristics that are shared by many genes and are generally stable throughout time. Both recognition sequences and functional proteins could be encoded by it (Morello and Breviario, 2008). Motif prediction enables us to assess the functional and structural classification of family members (Ahmad et al., 2022b; Ullah et al., 2022). Further, the examination of the phylogenetic tree and motifs revealed that the majority of *PbSNAT* in the same branch exhibited a comparable distribution of motifs (**Figure 5**). This finding further strengthens the evidence for their grouping in the phylogenetic tree. All PbSNAT proteins in the motif analysis results exhibited motif1 (**Figure 5**), which signifies the conserved domain of the SNAT family. Motif1 was conserved across all PbSNAT proteins. Additionally, our study examines how variations in *cis*-acting elements of genes directly impact their expression and differentiation. Transcription factors that respond to *cis*-acting elements are utilized to control gene expression (Pan et al., 2021; Sharif et al., 2026a). Analysis of *cis*-acting elements in pear *PbSNAT* genes revealed that the majority are associated with environmental stress and hormone responsiveness (**Figure 6**). Accordingly, most *PbSNAT* genes are implicated in stress response. Notably, over 50% of *PbSNATs* possess elements responsive to abscisic acid, salicylic acid, and MeJA. These findings suggest that ABA, SA, and MeJA serve as crucial signals for *PbSNAT* regulation. This also implies a potential role for MT in functioning synergistically with other phytohormonal pathways.

### 4.2 *PbSNAT* genes are differentially expressed across different tissues

Previous studies have established that SNAT genes are highly expressed in leaves, as demonstrated in species such as tobacco (*NtSNAT1/2*), *Zea mays* (*ZmSNAT1/3*), and *Hypericum perforatum* (*HpSNAT1/2*) (Yao et al., 2025). However, certain SNAT genes also exhibit high expression in other tissues, such as roots, flowers, or seeds. Expression analysis in flowering Chinese cabbage revealed contrasting tissue specificity for two SNAT genes. *BcSNAT1* expression peaked in leaves, while *BcSNAT2* was predominantly expressed in roots. Developmentally, both genes showed the highest transcript levels during the three-leaf and early bolting stages (Anwar et al., 2023). To investigate the potential variations in function between *PbSNAT* genes in pear, we analyzed their expression patterns in various organs and under normal growth conditions. The results exhibited diverse expression patterns among these genes. Regarding the expression in various organs, two *PbSNAT* genes were observed in the roots, stems, leaves, flowers, and fruits. This suggests that these genes could potentially influence both vegetative and reproductive growth. From *PbSNAT1* to *PbSNAT42*, expression was observed in almost all the tissues (**Figure 12**). The rest of the genes merely showed any expression in all the tested tissues. Since they are primarily engaged in MT biosynthesis (Zhou et al., 2021), we speculate the *PbSNAT* genes are key components of pear developmental biology.

### 4.3 Pear reproductive biology is altered by *PbSNAT* genes

The crop production is influenced by the projected advancement of two pivotal phases of reproductive biology, such as flowering and fruiting. Environmental stresses can disrupt metabolic and cellular processes, leading to negative effects on the growth of fruits and flowers (Su et al., 2021a; Sharif et al., 2022b, 2022a; Xu et al., 2023). The development of flowers and fruits is fundamentally governed by phytohormones, which regulate key processes including initiation, growth, patterning, and ripening (Su et al., 2021b; Jia et al., 2023a, 2023b). Our study focused on the elevated expression of several *PbSNAT* genes throughout various stages of fruit development in different pear cultivars. Throughout the various phases of fruit development, we identified numerous genes that had a significant degree of expression. The mRNA expression levels of the *PbSNAT1* gene showed a notable rise during the S1-S7 stages in all five pear cultivars that were studied (**Figure 13**). On the other hand, we observed a high abundance of SNAT and MT in the pericarp and pulp tissue (**Figure 15**). MT was found to increase the production of gibberellins (GA3 and GA4), which induce parthenocarpy in Starkrimson pears, according to research by (Liu et al., 2018). MT affects fruit development by modulating the signaling pathways of important growth hormones, including auxins, gibberellins, and cytokinins (Wei et al., 2022). Owing to the higher expression of *PbSNAT* genes and accumulation of SNAT and MT during the fruit developmental stage, it would be interesting to examine these biomarkers through functional characterization.

## 5 Conclusion

A total of 51 *PbSNAT* genes were located after searching the pear genome database. These genes were categorized into six groups by the phylogenetic analysis. The growth, reproduction, and stress biology processes were impacted by the GO and cis-acting components of the *PbSNAT* genes. The interacting protein study found that *PbSNAT*, in conjunction with other proteins, may regulate important reproductive and developmental processes. The expression of *PbSNAT* genes in different tissues demonstrated their role in growth-related activities. Their importance in regulating fruit developmental processes is further demonstrated by the quantification of SNAT and MT and the comprehensive expression of *PbSNAT* genes in five pear cultivars. While *in-silico* components and expression-based studies provide valuable insights into several aspects of *PbSNAT*, their relevance in fruit development and maturity has to be elucidated through functional characterization of specific genes, particularly *PbSNAT1*.

## Data Availability

The datasets presented in this study can be found in online repositories. The names of the repository/repositories and accession number(s) can be found in the article/supplementary material.
