# Correction: Genome wide identification of serotonin N-acetyltransferase (*PbSNAT*) gene family and their role in pear (*Pyrus bretschneideri*) fruit development

**DOI:** 10.3389/fpls.2026.1837915

**Published:** 2026-04-16

**Authors:** Chen Chen, Xiangyu Zuo, Aneesa Gul, Shunyan Chen, Ahmad Ali

**Affiliations:** 1 Shanxi Datong University, Medical School of Shanxi Datong University, Datong, China; 2 School of Traditional Chinese Medicine, Shanxi Datong University, Shanxi, Datong, China; 3 College of Coastal Agricultural Sciences, Guangdong Ocean University, Zhanjiang, China; 4 State Key Laboratory of Tropical Crop Breeding, Key Laboratory of Biology and Genetic Resources of Tropical Crops Ministry of Agriculture and Rural Affairs, Key Laboratory of Conservation and Utilization of Tropical Agricultural Biological Resources of Hainan Province, Institute of Tropical Bioscience and Biotechnology, Chinese Academy of Tropical Agricultural Sciences, Haikou, China

**Keywords:** bioinformatic analysis, fruit development, gene family, *PbSNAT*, pear

There was a mistake in **Figure 1** as published.

**Figure 1:**
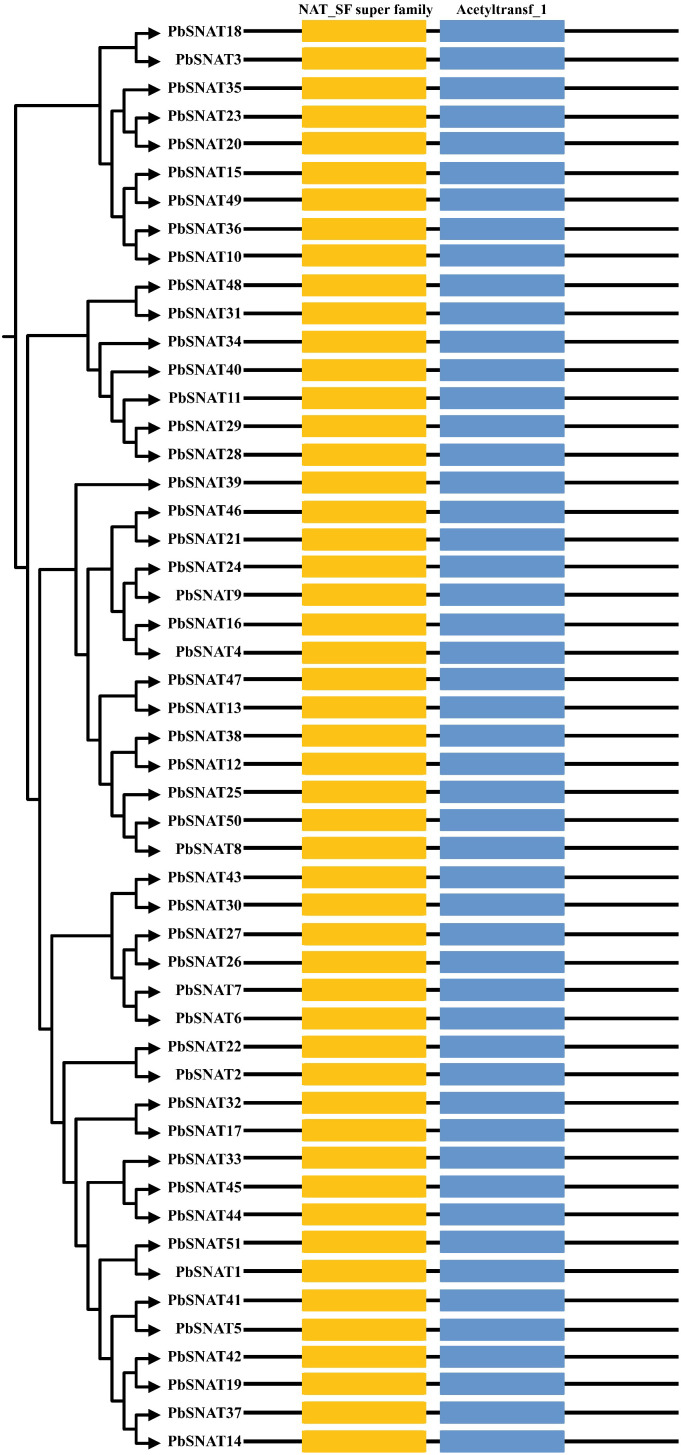
Schematic representation of the conserved domains of *PbSNAT* genes in *P. bretschneideri*.

**Figure 1** has been inadvertently duplicated within the figure panel. The corrected **Figure 1** appears below.

The original version of this article has been updated.

